# Spatiotemporal Identification of Potential Tsunami Vertical Evacuation Sites: A Case Study of Shizuoka City, Japan

**DOI:** 10.1371/currents.dis.e66442ce2b19de55532457d967d9645d

**Published:** 2017-04-24

**Authors:** Gerasimos Voulgaris, Jelena Aleksejeva

**Affiliations:** Department of Geoenvironmental Sciences, Graduate School of Life and Environmental Sciences, University of Tsukuba, Tsukuba, Ibaraki, Japan; Department of Geoenvironmental Sciences, Graduate School of Life and Environmental Sciences, University of Tsukuba, Tsukuba, Ibaraki, Japan

## Abstract

**Introduction:**

The city of Shizuoka directly faces the Nankai Trough (known for its tsunamigenic history), and is facing a potential tsunami threat. In this setting vertical evacuation can be of great significance in reducing loss of life.

**Methods:**

We apply a GIS based method in order to identify sites that could be utilized for vertical evacuation within the existing building stock of the city, under two tsunami scenarios of 5 and 10 meters of run-up. For each building, we estimate the volume that is expected to be lost per scenario, as well as the number of people inside and how that number fluctuates over different times of the day.

**Results:**

Using the criteria of 25% or less building volume loss and 6 cubic meters of volume per person, resulted in 2,046 potential sites for the 10 meter scenario and 1,643 potential sites for the 5 meter scenario, with the maximum amount of people that can potentially be accepted in these sites in the morning hours being 873,537 in the 10 meter scenario and 304,734 in the 5 meter scenario.

**Discussion:**

Our approach has shown that there is a temporal aspect in tsunami vertical evacuation due to the movement of the local population throughout the day. the proposed method can be used for preliminary identification of potential vertical evacuation sites, however, it must be followed by further vulnerability and engineering assessments of buildings, in combination with accessibility and evacuation routing in order to reach a viable and complete evacuation plan.

## 1. Introduction

Vertical evacuation is becoming increasingly considered in tsunami preparedness, after the impact of the 2004 Indian Ocean and the 2011 Tohoku tsunamis. Extensive publications from organisations such as FEMA and the Joint Research Centre (JRC) of the EU^1^^,^^2^, describe the concept of vertical evacuation and the characteristics of vertical evacuation sites. According to FEMA^1^, a vertical evacuation site is any building or high ground that can withstand an incoming tsunami and can accept incoming individuals who seek safety. Shibayama et al.,^3^ provide a similar description of what evacuation areas should be in Japan, classifying them into three categories of open high ground areas, buildings with more than 7 floors and buildings between 4 and 6 floors.

Despite these extensive guidelines, case studies concerning vertical evacuation have been somewhat limited. In contrast, research concerning tsunami evacuation in general, such as behavioural analyses (eg 5), evacuation simulation studies (eg 4), and research concerning the performance of structures during the Tohoku tsunami (e.g 6^,^^7^^,^^8^) have been extensive. When it comes to vertical evacuation studies have given insight on some of its aspects. For example, Park et al. 9 , provide a mathematical model of determining optimal locations for vertical evacuation sites in coastal areas with heavy focus on their spatial distribution. Raskin et al,^10^ performed an evaluation of buildings that could serve as evacuation sites, based on their engineering attributes, focusing mostly on public buildings such as government offices.

While the contribution of the above research is significant, there are still many aspects of vertical evacuation that have not been yet covered. One attribute, and perhaps among the most important ones, is the population that is affected in a potential tsunami flood-zone. To the best of hour knowledge, only Wood et al.^11^, have provided a spatial analysis of tsunami vertical evacuation shelters that includes local population as a basic attribute of the approach, and under the scope of a vertical evacuation plan. However, given the recent rise of "big data", population can now be approached not only as a static attribute, but spatiotemporally and on the microscale, in locations where such data is available.

We propose an approach that utilises and combines building inundation and temporal building population estimation, in order to identify existing urban structures as potential vertical evacuation sites. The objectives of our study and our used approaches are:

- Utilise variable tsunami scenarios
- Estimate the inundation ratio of structures in the study area
- Estimate the population of buildings in the study area and how it varies over the course of a day
- Establish criteria for potential vertical evacuation sites based on available and produced data
- Identify potential vertical evacuation sites and analyse their capacity to accept evacuees during different hours of a day

## 2. Study area selection and overview

To reach the objectives of this study, we sought to approach an area that is currently in high risk for tsunamis. The Nankai trough, is a subduction zone where the northern part of the Philippine plate subdues beneath southwest Japan resulting in high magnitude earthquakes 12. The trough has been extensively studied as per its seismicity and tsunami events. In historic times, earthquakes with magnitude of 8.0 or higher and a 120 year occurrence interval ,have happened in the area, with the most recent ones being the M 8.0 Tonankai earthquake of 1944 and the M 8.1 Nankaido earthquake of 1946 13. Tsunami deposits found along the extensive coastline facing the Nankai trough also attest to a tsunami occurrence interval every 100 to 200 years for the last 3000 years 14. The recorded activity of the trough implies further activity with an upcoming earthquake in the near future 15.

In this setting, we aimed to select a city which belongs in one of the 8 prefectures of Japan that directly face the Nankai Trough. Our choice became the city of Shizuoka, Shizuoka prefecture for two main reasons: (a) The city plays a very important role in Japanese geography. Not only it is the capital of the prefecture hosting a range of administrative functions, but it also is a central transportation node connecting north and south Japan. (b) It has excellent data availability compatible with the research objectives of this study. The most attractive data set is the people flow database which describes the population movement of the city over the course of 24 hours of the day.

The city of Shizuoka (Figure 1) directly faces the Nankai trough being in a vulnerable to an upcoming tsunami location. It is the second largest city in the prefecture with a population of 704,340 people 16. While the city boundaries greatly extent into the mountain ranges situated in the northern parts of the prefecture, these areas are uninhabited in their majority, and the populated built-up part of the city is mostly situated along the coastline. Three main highways and a two major train lines intersect the built up area of the city from the southwest to the northeast, dividing the city in two. The Central Business District (CBD) of Shizuoka is located to the northern part further away from the coastline, and a satellite business district is situated in the eastern area of Shimizu in very close proximity to the sea.

**Location of the city of Shizuoka, its three wards and its Central Business District (CBD):**
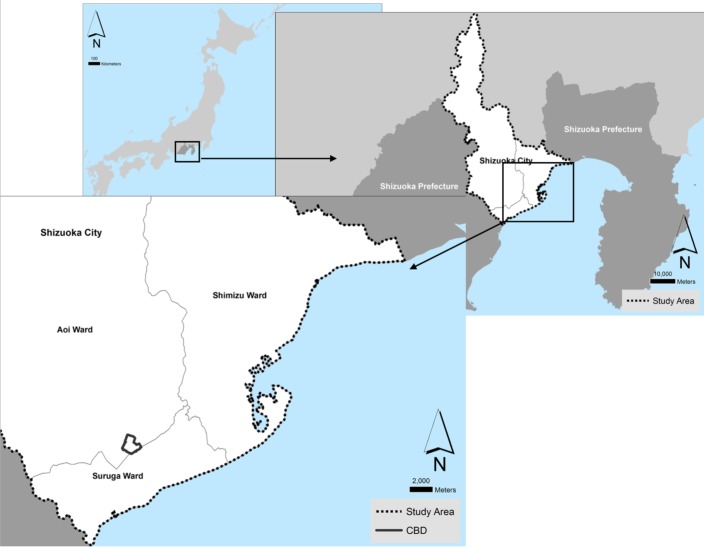

## 3.Tsunami scenarios and building inundation ratios

**3.1 Scenario Selection**

Despite the numerous studies that illustrate the seismic and tsunami presence along the Nankai Trough, clear descriptions of the characteristics of past remains remain limited. Sugimoto et al. 17 describe that the 1707 Hoei Earthquake, which originated in the Nankai Trough, was estimated to have had a magnitude of 8.4, while Imai et al. 18 suggest that the tsunami that followed the earthquake reached a maximum run-up of 5 meters.

Beyond that, characteristics of potential tsunamis from earthquakes of such magnitudes have to be inferred indirectly. For example, most tsunami magnitude scales (e.g. 19) suggest that tsunamis from magnitudes in the range of 8 should be expected to have a maximum run-up of 10m. Under these circumstances, it becomes clear that any method of identifying potential vertical evacuation sites should offer flexibility and adaptability to different scenarios originating from different sources.

We used two tsunami scenarios in our approach, the 5m run-up one as described by Imai et al. 18, as well as what the tsunami magnitude scales suggest, a 10m run-up scenario. Using GIS, we were able to visualise both scenarios using a Digital Elevation Model for the city of Shizuoka with a resolution of 5m, as it was provided by the Geospatial Authority of Japan (GSI). Subtracting the elevations from the maximum run-up resulted in water depth maps as can be seen in Figures 2and 3.

The 5m scenario affects only small areas of the coastline of the city, with most of the flooding occurring in the area of Shimizu to the North. However, the 10m scenario shows greater capacity for flooding, as not only it affects the majority of the city's coastline, but also, it follows the river system in Shimizu uphill, achieving greater inland penetration.

**10 meter run-up tsunami scenario for the city of Shizuoka:**
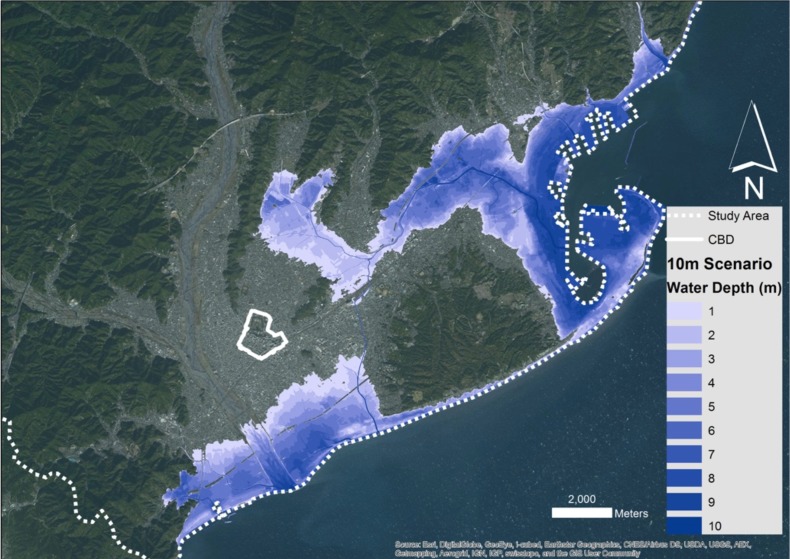

**5 meter run-up tsunami scenario for the city of Shizuoka:**
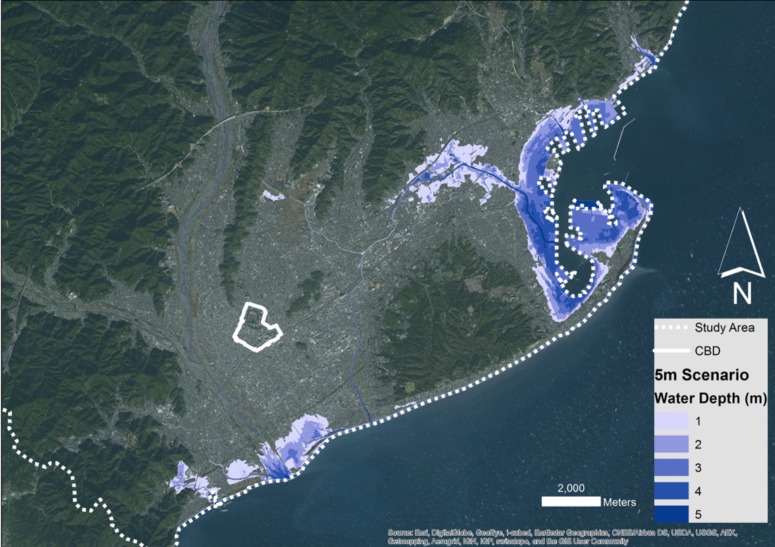

**3.2 Inundation ratio estimation**

Continuing with our GIS approach, we utilised a process of estimating the extent that buildings become inundated under a tsunami. The Inundation Ratio estimation is a widely used approach, usually in studies that investigate the vulnerability of buildings to tsunamis (e.g.^20^^,^^21^). As building heights are necessary for this estimation, we had to obtain a dataset that contains such information for the full building stock of the city. The best choice that offered coverage for the full extent of Shizuoka was the Zenrin Zmap Town II dataset which contains floor number information for each individual building. We had to assume that each floor was 3 meters in height, and as such, we indirectly obtained the necessary building heights. The dataset also contained the information of each building's footprint geometry, which in combination with the height, allowed us to finally have an estimate of each building's volume.

As a final step, the building volume and the water depth at the location of each building were used to estimate the percentage of the building's volume that becomes flooded under both the 5m and the 10m scenarios as follows:

\begin{equation*}VA=VB - VB(EX-H)\end{equation*}

Where VA is the available volume after the tsunami, VB is the volume of the building, EX is the exposure, or water depth at the location of the building and H is the building height.

The results of this estimation can be seen in Figures 4 and 5, where the individual building volume loss to the tsunami is mapped. This process gave two important products for our study. First, the volume that is available in buildings after the tsunami could be utilised by people for evacuation within the flood zone. Moreover, there is a distribution of buildings with such available volumes across the full extent of both the 5m and 10m scenario flood zones.

**10 m run-up scenario building volume loss:**
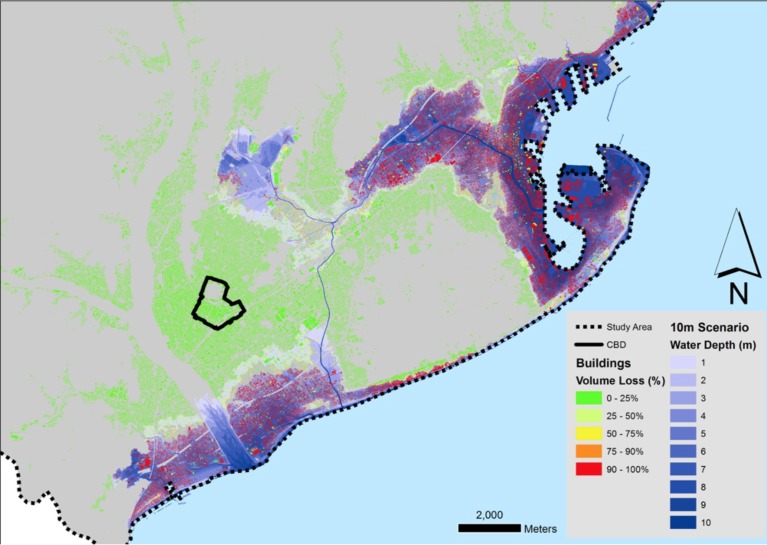

**5 m run-up scenario building volume loss:**
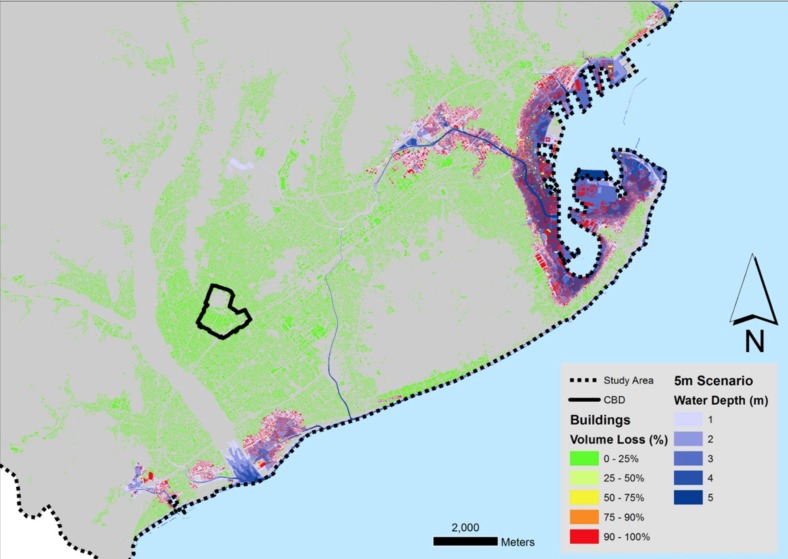

## 4. Temporal building population estimation

**4.1 Background and Method Selection**

For decades now it has been identified that regardless of the detail of a population census, what is often reflected by its statistics is known as nighttime population (e.g.^22^), meaning that spatially, the census shows where the people will most likely be situated at night when asleep at their homes. However, in reality, the population of a populated area is mobile during the course of a day, and can be distributed in numerous locations at the time when a tsunami occurs. When it comes to the Japanese census, the Statistical Agency of Japan, provides extensive datasets that despite their detail still cannot reflect the mobility of the population. Moreover, even when considering the nighttime population itself, the spatial distribution of the population cannot go lower than 500 m, due to respect of the privacy of Japanese citizens.

The second variable to our potential vertical evacuation site identification would be the number of occupants inside a building and by extension how much volume they occupy. As such, the information provided by the census was somewhat limited and additional estimations were necessary in order to not only achieve a spatial resolution of building level, but also, the temporal distribution due to the mobility of the population. Lwin and Murayama 23 had previously introduced a method of estimating the population of buildings in an area, based on census grids and the volume of buildings. The authors themselves promote this method for its usefulness in natural disaster studies and we previously have been successful in incorporating its results in tsunami vulnerability assessments 24. While this method provides the required spatial distribution, it still lacks the necessary temporal dimension.

Greger 29, used Lwin and Murayama's method as a base and proposed a spatiotemporal building population estimation method. The way he achieved that was by introducing additional datasets to the process that were available for his study area in Tokyo. The first dataset is the spatial distribution of the workforce, in 50 m resolution as it is described by the census. The second, was a point address dataset that allowed him to determine the use of each building, and the third was the PersonFlow data for Tokyo, as provided by the Center of Spatial Information Science University of Tokyo 25. By using the results described in Axhausen et al.^26^, Bowman and Ben-Akiva 27 and Jiang et al.,^28^ he defined six activity types and matching building usages. By combining the movement of people, the census, and the building categories, he was able to estimate the population of people in buildings and how it varies over 24h of a day, using the following equation:

\begin{equation*}BP_{i,c,t}=\left( \frac{AP_{A_{i,c,t}}}{\sum_{k\in A_{i,c}}{BA_{k}\cdot BF_{k}}} \right) \cdot BA_{i,c}\cdot BF_{i}\end{equation*}

Where BPi,c,t is the building population of building i in category c at time t, APAi,c,t is the total population of category c at time t of the census tract that contains building i, A is the set of all census tracts, BAi is the footprint area of building i, BAi,c is the footprint area of building i in category c, and BFi is the number of floors of building i 29.

Greger's method has great compatibility with the datasets available for Shizuoka, which led to its selection for our temporal building population estimation process. While the data available for Shizuoka were similar to the ones Greger utilised for Tokyo, there were some differences that led to small adjustments to the application of the method.

**4.2 Method application**

Figure 6 presents the datasets that we utilised for the 24 h building population in the city of Shizuoka. All of these except one were consistent with the data demands of Greger's method. The dataset that was inconsistent was the point address dataset of February 2014 that we used could not match the six categories of activities that Greger suggested for Tokyo. The only possible distinction was whether each building was a residence or not. Because of this limitation we had to adjust buildings into two categories, residences and workplaces. Moreover, the movement of the population during the course of a day had to be grouped into two movement types to match the building categories as follows : to home and to work (Figure 7).

**Datasets that were used for the purposes of this study:**
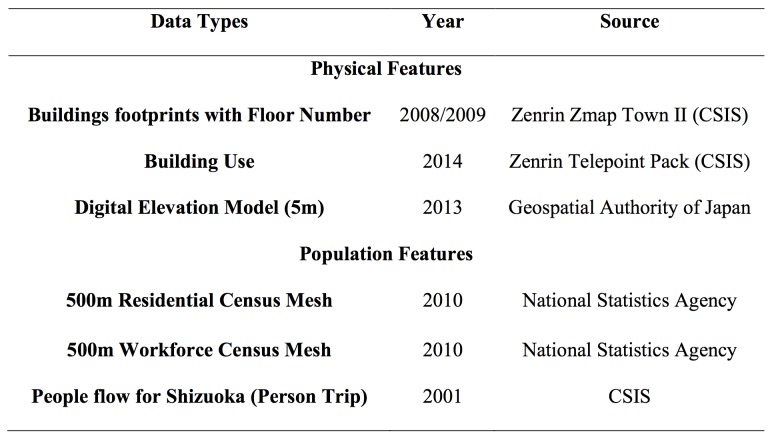

**People flow movement over the course of the day grouped in three categories. Only the categories of Home and Work were considered. Data source: CSIS:**
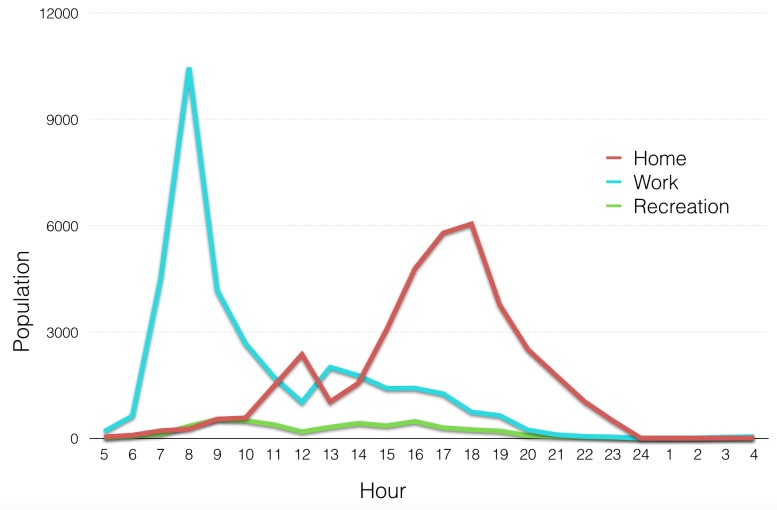

Applying the method resulting in two datasets. One for building footprints of residences and one of workplaces, both of which contain the estimated population of each building in 24 hourly segments. At the end of this step we had obtained the information necessary for our identification of potential vertical evacuation sites: (a) The volume available to be occupied in each building after two tsunami scenarios, and (b) the estimated population of each building at each hour of the day (Figure 8).

**Results of the 24 hour building population estimation method for the city of Shizuoka at 12pm:**
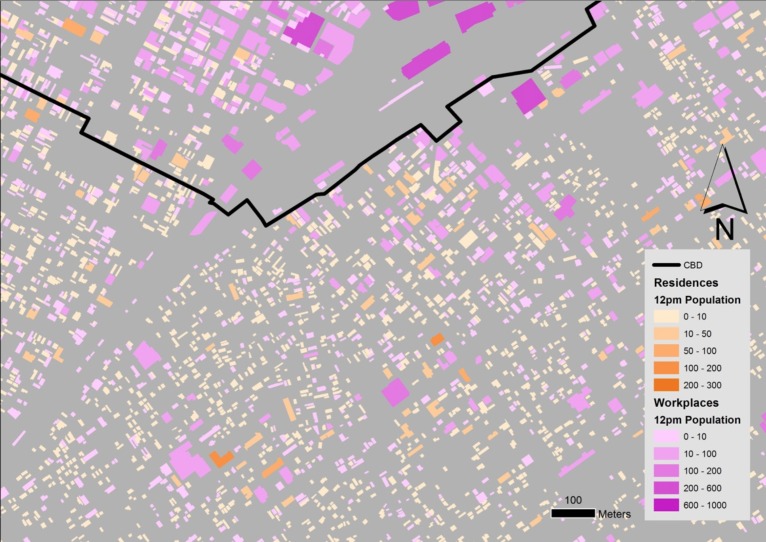

**4.3 Validation**

We opted to validate the building population estimation method with field measured data, not only to verify the method itself, but also its slight adjustment to the datasets for Shizuoka City. We followed the same method as Greger 29, by observing the number of individuals that exit and enter two workplace buildings, one in the centre of Shizuoka and one in the are of Shimizu. Out of respect to the local residents' privacy, we did not engage in any measurements of residential buildings, and the exact location of the workplaces will selected will also be kept under discretion.

Figures 9 and 10, show the field observed populations for the two buildings and the estimated by the method populations. Building A, an office building has similar estimated and observed values, however, in the late evening hours where the method estimated that people would be returning home, in reality there was a social gathering a number of people as well as some visitors remained in the building till later hours. Given the nature of the data used, we anticipate that it would be of extreme difficulty for this method to anticipate for such irregularities. Building B, was a bank, and similarly to building A the method has similar observed and estimated population values, except for some differences that are due to increased client numbers at different hours. Once again, such variances can be completely random and isolated and would be extremely difficult to be captured by any dataset, and consequently a method.

Excluding the small irregularities that were observed, the majority of measured and estimated populations were of similar values and we were able to verify a close approximation of the population of buildings by the applied methodology.

**Observed and estimated populations values for Building A, an office building in the area of Shimizu:**
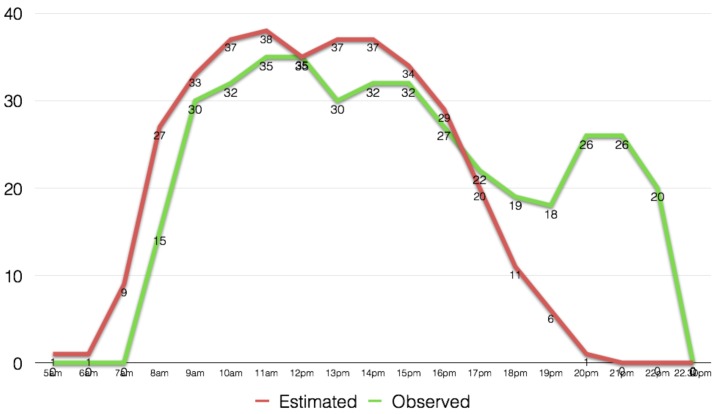

**Observed and estimated populations values for Building B, a Bank in the CBD area of Shizuoka:**
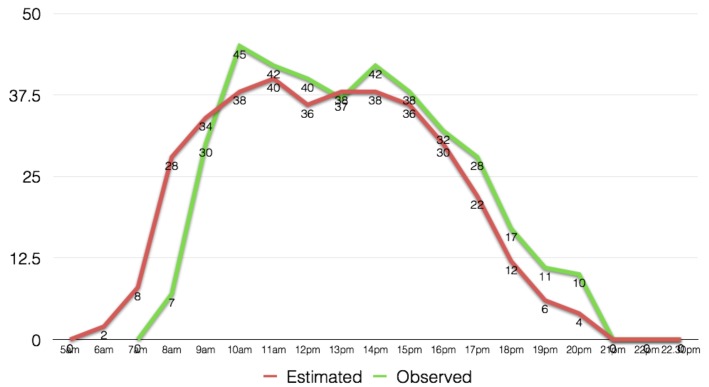

## 5. Potential vertical evacuation site identification

**5.1 Evacuation Criteria**

In the literature review section it was discussed how vertical evacuation studies in the past have been limited, and how the main focus of the majority of studies has been the physical characteristics of buildings such as their resilience, shape and height. Because of that, there is an absence of established criteria on how populated or unpopulated a building has to be in order to be used for vertical evacuation, and by extent how much volume an evacuee would occupy in that building.

Our concept for this approach is that each building that is more than one floor and is not completely flooded can provide its available volume for vertical evacuation. Moreover, we anticipate, and have demonstrated by our building population estimation, that the volume available for evacuation is expected to be variable during different times of the day as such buildings are expected to be occupied by individuals either for residence or work. In the absence of a previous framework of necessary volume standards we introduced plausible criteria to test our approach. It should be noted that due to the nature of the method these criteria can be adjusted or updated with any other criteria necessary by a complete vertical evacuation plan.

In order to complete the identification of potential vertical evacuation sites we assumed the following: (1) Potential vertical evacuation sites will be buildings with more than one floor, and have an inundation ratio of 25% or less. This ensures that even buildings with two floors are not going to be flooded to extents that might potentially destroy them. (2) Each individual that is either situated in the building already, or is going to evacuate to the building is going to occupy 6 cubic meters of volume. The volume required by those already situated inside is going to be subtracted from the available volume and the rest will be used to determine how many additional individuals can be accommodated for vertical evacuation. Finally, at a given hour of the day X, the number of people that can be accepted in each building that is 25% or less flooded was estimated by the following equation:

\begin{equation*}N=\frac{(VB-IR)-(PBX\times VE)}{VE} \end{equation*}

Where N is the number of people that can be accepted by the building, VB is the volume of the building, IR is the inundation ratio of the building, PBX is the population of the building at time X, and VE is the volume allocated per person for evacuation.

**5.2 Results**

Using the above formula for both scenarios produced two kinds of results. The first, is the total number of potential vertical evacuation sites per scenario, and the second, the number of people that can be accepted in each building and in the whole of the flood zone of each scenario.

For the 10m scenario, there were 2,046 buildings that met the criteria, out of which 1,551 were residences and 410 were workplaces. In the case of the 5m scenario, 1,643 buildings were identified, out of which 1,233 were residences and 410 were workplaces. Concerning the population that can be accepted in these buildings, the results showed that the number was variable during different times of the day. Figure 11 summarises the total number of people that can be accepted in these potential sites, for each scenario and for different hours of the day. We found that as the population moves, there are discreet differences in the available volume that buildings can offer to evacuees. The morning hours between 10 and 11 offer the most available volume and show the highest capacity for evacuees. In the case of the 10 m scenario, up to 873,537 individuals could be accepted, while in the 5 m scenario the number was down to 304,734. This translates to more than 100% of the population of the city for the 10m scenario and to 43% of the population for the 5 m scenario. The 5m scenario shows a much lesser capacity to accommodate evacuees, however, given the much limited extend of its respective flood zone, the potential for vertical evacuation is still high. The total capacity for evacuees lessens as the day progresses towards the evenings with the late night hours having the smallest numbers of vertical evacuees that can be accepted.

**Temporal variations of additional population that can be accepted for vertical evacuation in buildings 25% or less inundated:**
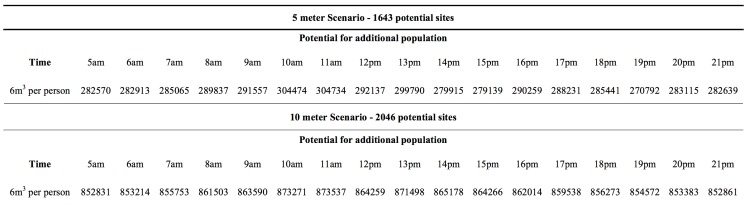

**5.3 Discussion**

The results that were described earlier demonstrate that there is a difference between the two scenarios when it comes to the number of potential sites as well as the number of individuals they can support. This can be attributed to the fact that the severity between the two scenarios is different, and they have flood zones that have variable extents and not only affect different number of buildings but also numbers of populations that are situated in these locations.

In both scenarios the buildings that were identified as potential vertical evacuation sites were residential as well as workplaces, but the residences outnumbered the workplaces 3 to1 and 4 to 1 in the 10 m and 5m scenario respectively. This introduces the temporal dynamic that was verified by the fact that the number of people that can evacuate in the potential sites is increased in the morning hours and decreased in the evening and night hours. Considering that in both scenarios we have many more residences than workplaces as potential vertical evacuation sites, could explain the higher available volumes in the morning, as many locals leave their residences to engage in daily activities, and move to different parts of the city. In contrast, the evening hours have less available volume as the residents return home occupying these previously less populated buildings, while the workplaces that are now scarcely populated or empty are not high enough in numbers to match the morning volumes of residences.

Despite these differences during different hours of the day, the population that can fit in these potential sites was surprisingly high. In fact, within the flood zone of the 10m scenario there is enough volume available in potential sites to accommodate the whole population of the city of Shizuoka in the morning hours. At the same time, in the limited flood zone of the 5 m scenario, there is still enough volume for 43% of the population of the city. This could mean that there is an abundance of volume in the buildings of Shizuoka, with some of them being empty or scarcely populated. Observations from field work in the city pointed towards this direction, but no qualitative methods were used to verify them.

It should be stressed that the buildings identified by our approach are sites that can be potentially used for vertical evacuation. In this case we focused on the inundation and perhaps mainly on the population of those buildings. Due to the extent of the flood zones and the number of buildings in Shizuoka we were unable to incorporate structural attributes into our method. The results produced in this study should be used as part of a greater vertical evacuation plan, which can offer the appropriate resources for a structural evaluation of those buildings. Moreover, access to the potential sites, as well as the general procedures of the evacuation itself remain outside the scope of this study due to similar limitations, and should be the focus of further research or evacuation planning in the future.

## 6. Conclusions

Our approach has shown that the attributes of building population and tsunami severity affect the way buildings can serve as vertical evacuation sites in a spatiotemporal way. The inundation of buildings differs between the 5 meter and 10 meter scenarios not only number of buildings within their flood zones, but also in the way buildings are extensively flooded or not in overlapping areas of the scenarios, indicating discreet spatial variations.

Using Greger’s^29^ building population estimation method has revealed a temporal aspect of vertical evacuation due to the mobility of Shizuoka’s population over the course of a day. Residential buildings were shown to be emptier during the daytime while non residential workplace buildings became more populated, with the opposite occurring in the evening, night and early morning hours.

The number of individuals situated in buildings at a different hour dynamically affected the potential of acceptance of additional individuals that are vertically evacuating. By setting criteria of buildings inundated to 25% or less of their volume, and 6 cubic meters of volume per person we showed that for the case of Shizuoka City, its whole population could potentially fit in the available building volume in the case of the 10 m scenario and 43% of the city’s population in the 5 m scenario.

The tsunami inundation scenarios and vertical evacuation criteria used to test our approach can be exchanged for any kind of scenario and criteria that match any goals or research pursuits. Moreover, the method can be applied in other areas of Japan or the rest of the world, where similar datasets are available, or can be obtained by field work. However, the results of our method identify sites that can only potentially be considered. Vulnerability and engineering assessments are necessary in order for the viability of these sites to be concluded. Accessibility and evacuation routing to these sites must also be considered, under the scope of a complete evacuation plan.

## Competing Interest Statement

The authors have no competing interests.

## Corresponding Author

Gerasimos Voulgaris (gvsquall@gmail.com)

## Data Availability Statement

All relevant metadata and resulting data are within the article. Open source datasets used were obtained from the following two institutions: (a) Geospatial authority of Japan (GSI). In their website, free registration is required for open access to GIS data for the whole of Japan. Visit http://fgd.gsi.go.jp/download/menu.php and select the dataset option required. They are divided in basic map information and Digital Terrain Models. Upon selecting the required data, free registration is required before finalising the download. (b) The National Statistics Bureau of Japan. While their website has English support it might be limited. To obtain any kind of spatial statistics, visit http://e-stat.go.jp/SG2/eStatGIS/page/download.html , then select the area and type of statistics required and proceed to download. Minimal personal information such as institution and purpose might be required. Non open source datasets were obtained via the Joint Research Assist System (JORAS) of the Center of Spatial Information Science (CSIS), Tokyo University. In order to obtain data, application via form is necessary at CSIS. By visiting http://www.csis.u-tokyo.ac.jp/english/joint_research.html , and completing the relevant form, it is possible to obtain the relevant datasets for non commercial use only.
